# Cellular Transformation by Human Cytomegalovirus

**DOI:** 10.3390/cancers16111970

**Published:** 2024-05-22

**Authors:** Georges Herbein

**Affiliations:** 1 Department Pathogens & Inflammation-EPILAB EA4266, University of Franche-Comté (UFC), 25000 Besançon, France; georges.herbein@univ-fcomte.fr; 2 Department of Virology, CHU Besançon, 25000 Besançon, France

**Keywords:** HCMV, cytomegalovirus, oncoviruses, oncogenesis, oncomodulation, hallmarks of cancer, PGCCs, high-risk HCMV

## Abstract

**Simple Summary:**

Discovering new oncoviruses is a main goal of virology research. In addition to its deleterious role in immunocompromised patients and during pregnancy leading to birth defects, the human cytomegalovirus’s (HCMV) potential role as an oncogenic agent has garnered significant attention recently. This perspective article focuses on the transforming potential of HCMV based on recently unveiled molecular and cellular characteristics of HCMV-infected cells.

**Abstract:**

Epstein–Barr virus (EBV), Kaposi sarcoma human virus (KSHV), human papillomavirus (HPV), hepatitis B and C viruses (HBV, HCV), human T-lymphotropic virus-1 (HTLV-1), and Merkel cell polyomavirus (MCPyV) are the seven human oncoviruses reported so far. While traditionally viewed as a benign virus causing mild symptoms in healthy individuals, human cytomegalovirus (HCMV) has been recently implicated in the pathogenesis of various cancers, spanning a wide range of tissue types and malignancies. This perspective article defines the biological criteria that characterize the oncogenic role of HCMV and based on new findings underlines a critical role for HCMV in cellular transformation and modeling the tumor microenvironment as already reported for the other human oncoviruses.

## 1. Introduction

Human cytomegalovirus (HCMV) (also named HHV-5) belongs to the herpesvirus family, is an enveloped double-stranded DNA virus, and is ubiquitous with no seasonal variations [1]. Although usually asymptomatic in healthy individuals, HCMV infection can result in severe CMV disease in immunocompromised subjects and lead to congenital infections with severe neurological sequelae including sensorineural hearing loss in the pediatric population [2]. Recently the role of HCMV in cancer has been re-evaluated.

Several years ago a first paradigm named oncomodulation was forwarded to explain the role of HCMV in cancer where the virus will accelerate the transformation process in infected tumor cells. Although the high prevalence of HCMV in several tumors has been reported [3], it is difficult to know whether the presence of HCMV is incidental due to the viral infection on top of an already present tumor or if the HCMV by itself can start and favor the cellular transformation, and thereby could be defined as a genuine oncovirus [4]. Since stem cells are permissive to HCMV and the appearance of cancer stem cells (CSCs) is a main player in tumor initiation and tumor spread it’s worth questioning the genuine transformation potential of HCMV.

Epstein–Barr virus (EBV), Kaposi sarcoma human virus (KSHV), human papillomavirus (HPV), hepatitis B and C viruses (HBV, HCV), human T-lymphotropic virus-1 (HTLV-1), and the most recently discovered Merkel cell polyomavirus (MCPyV) belong to the group 1 carcinogens for humans, namely human oncoviruses [5,6]. Most oncoviruses transform cells through viral oncoproteins or activation of cellular oncoproteins. In addition, oncoviruses such as HCV favor neoplasm development mainly by chronic inflammation. The hallmarks of cancer were proposed as a set of functional capabilities acquired by human cells as they make their way from normalcy to neoplastic growth [7], and were twice updated in 2011 and 2022 to include additional biological factors [8]. Human oncoviruses fulfill the hallmarks of cancer [9], and recently HCMV has been recognized to display all the hallmarks of cancer as defined in 2011 [10].

Findings by our group and others indicated that HCMV displays oncogenic properties, in addition to the already reported oncomodulatory effect [3,4,10,11]. We will focus the present perspective article on the similarities between HCMV and the already described seven human oncoviruses, HCMV’s immunosuppressive impact on the tumor microenvironment, and the fulfillment of the recently reported hallmarks of cancer by HCMV-transformed cells.

## 2. HCMV Displays Oncogenic Traits Similar to Human Oncoviruses

In addition to the already recognized seven human oncoviruses, several points are in favor of a direct oncogenic role for HCMV [3,4,12,13,14,15] (Table 1). The stem cells are permissive to HCMV with Thy-1 and platelet-derived growth factor receptor alpha (PDGFRα) identified as stem cell markers that favor HCMV infection [16,17,18,19]. HCMV could favor oncogenesis through the infection of stem cells, the generation of cancer stem cells, and/or the dedifferentiation of mature cells toward stem cells or progenitor cells. HCMV can alter and impair DNA repair pathways parallel to enhanced cell survival [20,21]. Among the viral proteins, the immediate early protein 1 (IE1) favors stemness and EMT in glioblastoma cells [22,23,24]. We reported recently that the influence of oncoviruses and HCMV on Myc and EZH2 expression could be a key driver of cellular transformation. Oncoviruses and HCMV can modulate the levels of Myc and EZH2, two key oncogenic/stemness players, through various molecular mechanisms including among others cell cycle dysregulation, epigenetic modifications, apoptosis blockade, increased cell proliferation, and the generation of polyploid giant cancer cells (PGCCs) [25,26].

The alternance of lytic and latent phases occurs during the pathogenesis of oncoviruses of the Herpesviridae family, namely EBV and KSHV, with viral reactivation triggered by immune suppression and inflammation [70,71,72]. Similar to EBV and KSHV, in such a moving immune environment, HCMV variants emerge and HCMV fitness could be favored with the distribution of the virus between distinct anatomical compartments [73,74,75,76,77,78,79,80,81,82]. The diversity of HCMV strains could play a role in cellular transformation, although cancer-derived cell lines are not always fully permissive to HCMV replication [83,84,85,86,87]. In addition, very low levels of HCMV replication could be at play in the transformation process [88,89]. In fact, we isolated HCMV clinical strains in our laboratory, namely HCMV-DB and BL, which fully replicate in human mammary epithelial cells (HMECs), ovarian epithelial cells (OECs), prostate epithelial cells (PECs), and astrocytes followed by their transformation with the appearance in cultures of CMV-transformed HMECs (CTH cells), CMV-transformed OECs (CTO cells), CMV-transformed PECs (CTP cells), and CMV-elicited glioblastoma cells (CEGBCs) [60,61,62,63,66,67,68,69]. The HCMV-DB and BL strains were named high-risk oncogenic strains by the author (HR-HCMV) [90]. HR-HCMV strains were latent and/or replicated at low levels in chronically infected transformed cells and were reactivated following 12-O-tetradecanoyl-phorbol-13-acetate (TPA) treatment [62,67], similar to KSHV and EBV reactivation under TPA [91,92]. We believe that the latency phase is an important player in the transforming process of epithelial cells infected with HR-HCMV since in chronically infected transformed cells, we detect very low levels of HCMV with reactivation by MIEP activators such as TPA or HDAC inhibitors. In addition, in these transformed cultures, we observe time-by-time spontaneous transient “viral blips” indicating that transient lytic phases occur among the latently infected cells. Following the infection of epithelial cells and astrocytes by HR-HCMV, cellular dedifferentiation parallel to cancerous traits was present in CTH cells, CTO cells, CTP cells, and CEGBCs and could ultimately lead to the appearance of adenocarcinoma of poor prognosis such as triple-negative breast cancer, high-grade serous ovarian cancer (HGSOC), prostate cancer, and glioblastoma [62,63,64,65,67,69]. In addition, the direct role of HCMV in the appearance of glioblastoma is likely with the detection of the virus in all the glioblastoma biopsies tested in our group [66], similar to more than 99% of HPV DNA detection in cervical carcinoma [93]. Although many groups have confirmed the presence of HCMV in glioblastomas, others could not. We cannot exclude that optimized immunohistochemistry and PCR techniques are required to detect it (reviewed in [15]). Recently our group reported that human astrocytes chronically infected and transformed by oncogenic HR-HCMV strains (HCMV-DB strain and HCMV strains isolated from glioblastoma patients) generated spheroids that resulted in the appearance of glioblastoma-like tumors in xenografted mice [94]. The direct oncogenic role of HCMV following acute infection of primary epithelial cells (HMECs, OECs, and PECs) and human astrocytes is summarized in Table 2.

## 3. HCMV, like Human Oncoviruses, Favors an Immunosuppressive Tissue Microenvironment

Oncoviruses impact the TME with a chronic inflammatory environment and altered signaling of cell–cell and cell–extracellular matrix adhesion molecules that favor the spread of cancerous cells and metastasis [95]. TME is characterized by the presence of tumor-infiltrating myeloid cells, including tumor-associated macrophages (TAMs), myeloid-derived suppressor cells (MDSCs), tumor-associated dendritic cells (TADCs), tumor-associated neutrophils (TANs), and cancer-associated fibroblasts (CAF). These cells display pro-tumorigenic activities with reactive oxygen species (ROS) production, modulation of inflammation, tumor progression, and angiogenesis; this could be fueled by oncoviruses [96]. Oncoviruses and HCMV along with EZH2 and Myc play a crucial role in shaping an immunosuppressive TME (reviewed in [25]). Similar to oncoviruses, HCMV infection leads to an immune-tolerant environment that will favor tumor appearance, growth, and spread parallel to increased viral fitness [10,97,98,99]. Critical viral players among others are the IE1/2 and pUS28 proteins known for favoring cell survival and sustained cell transformation [4]. HCMV favors tumor cell survival and promotes its own fitness, and HCMV blocks the apoptotic machinery within infected cells through IE2, pUL36, and pUL37 [10]. HCMV decreases viral-specific CD4+ and CD8+ T-cell response and NK activity through several viral proteins (pp65, vIL-10, gpUL40, pUL16, pUS18, and pUS20), which could favor tumor growth [10]. A bidirectional relationship between tumor cells and HCMV could be at play which will curtail viral replication and favor tumor escape with a limited deleterious accumulation of the inflammatory cells at the viral infection site [10,100,101,102]. In addition the production of immune-suppressive cytokines such as cellular IL-10, viral IL-10 and TGF-beta will favor the appearance of TAMs which will further accelerate the tumor spread [103]. HCMV infection profoundly modifies macrophage identity rewiring specific differentiation processes, favoring viral spread and curtailing innate tissue immunity [104]. Interestingly, the first high-risk HCMV strain identified in our group, namely HCMV-DB, is highly macrophage-tropic, favors an M1 to M2/TAM shift upon infection, and leads to the appearance of transformed cells (CTH, CTO, CTP, and CEGBCs) in culture [4,60,105].

## 4. HCMV Fulfills Previous and Current Hallmarks of Cancer

**Figure 1:**
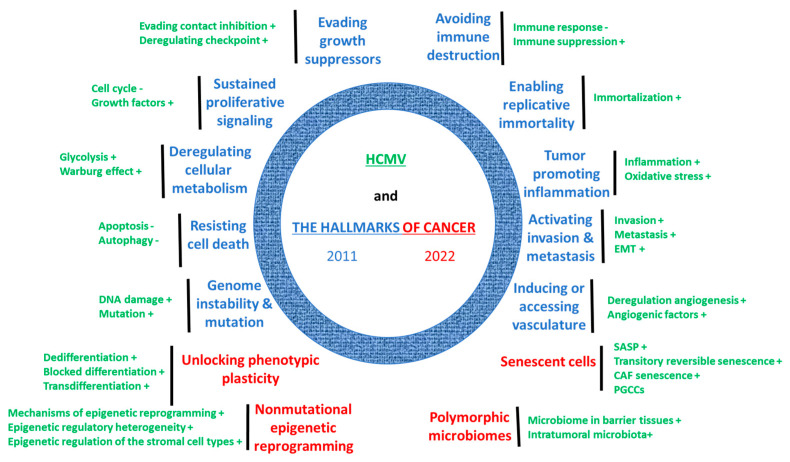
HCMV (high-risk strains) fulfills all the hallmarks of cancer described by Hanahan in 2022 [8].

We previously reported that HCMV infection fulfills all the hallmarks of cancer as defined by Hanahan and Weinberg in 2011 [106] (Figure 1). Recently, Hanahan revisited the hallmarks of cancer and added the four following hallmarks: unlocking phenotypic plasticity, nonmutational epigenetic reprogramming, polymorphic microbiomes, and senescent cells [8].

Dedifferentiation depicts the phenotypic plasticity of our in vitro model with HCMV’s transformation of HMECs, OECs, PECs, and human astrocytes [60,62,66,67,69]. Luminal-to-basal transition upon oncogenic stress activation was described [107], highlighting the epithelial compartment plasticity during tumorigenesis [108], where differentiated luminal epithelial cells can revert into functional basal stem cells in vivo [109]. Further, HMECs’ dedifferentiation into the malignant progenitor-like phenotype can be triggered by the transcription factor special AT-rich binding protein-2 (SATB2) [110]. This conceptualizes that development is a bidirectional process [111] and that somatic cells can gradually dedifferentiate into primitive stages of the developmental hierarchy, where HCMV, in our model, could be a dedifferentiation vector. Similar to the dedifferentiation of mature HMEC infected with HCMV-DB and BL strains, we observed dedifferentiation of mature ovarian epithelial cells and prostate epithelial cells into immature progenitor cells with stemness traits [67,69]. Interestingly both high-risk HCMV-DB and BL strains dedifferentiated primary mature human astrocytes into neural-progenitor-like tumor cells similar to the cells detected in glioblastoma, especially the ones adopting the Lévy-like movement patterns [66,112]. 

Nonmutational epigenetic reprogramming is present in HCMV-transformed cells parallel to stemness, dedifferentiation, and polyploidy [4,10,113]. HCMV-transformed cells display deregulated p53 and Rb pathways parallel to increased Myc expression [12,114,115]. The enzymatic subunit of polycomb repressive complex 2 (PRC2), EZH2, a histone-lysine N-methyltransferase responsible for transcriptional silencing [116], is increased in CTH, CTO, CTP, and CEGBCs cells [64,66,67,69], as reported in several cancers of poor prognosis [117,118,119,120,121,122,123,124]. All in all, a nonmutational epigenetic reprogramming occurs in HCMV-transformed cells namely through EZH2 upregulation, a downstream target of the Myc oncogene [125,126].

The evidence is increasingly compelling that polymorphic variability in the microbiomes between individuals in a population can have a profound impact on cancer phenotypes [127,128]. The microbial constituents of microbiomes likely will influence the progression of the cancers caused by oncoviruses. In fact, most people infected with oncoviruses will never develop cancer, meaning that other factors including the microbiome could favor transformation. For example, in a study of women with high-risk HPV infections and high cervical cancer rates, significant bacterial and fungal profile changes were associated with cervical squamous intraepithelial lesions and HPV infections [129]. In addition, HCMV similar to most of the human oncoviruses, e.g., HPV, EBV, and KSHV, shows viral strain variability with distinct oncogenic potential that could be further enhanced by the tumor microbiome [130]. In agreement with this observation, our data indicate that the oncogenic potency of HCMV clinical strains varies between low- and high-risk strains [60,62,63,90]. Only the high-risk HCMV strains can trigger the appearance of PGCCs [90]. Recently, the essential role of intestinal microbiota in murine cytomegalovirus reactivation was reported [131], suggesting a potential role for the microbiota in the alternance of latent and lytic viral stages, thereby fueling CMV diversity. Therefore, it will be critical in the future to assess the microbia present in breast, prostate, and ovarian cancers in light of the HCMV strains (especially high-risk) detected in tumors and to decipher the exact role played by the microbiota in HCMV diversity parallel to cellular transformation.

Although the protective effects of senescence in limiting malignant progression have been reported [132,133], more recently, it has become clear that senescent cells stimulate tumor development and malignant progression [133,134]. Senescence favors transformation and tumor growth through the generation of a senescence-associated secretory phenotype (SASP) with the release of cytokines, chemokines and proteases [135,136,137], and transitory and reversible senescent cell states whereby senescent cancer cells can escape senescence and resume cell proliferation and acquire oncogenic traits [138,139]. Polyploid giant cancer cells (PGCCs) have been recently reported as a major player in cancer [140,141]. PGCCs exhibit features of senescent cells [142], questioning the role of senescence in the generation of PGCCs observed in infections with oncoviruses [26]. Although non-oncogenic viral infections (human respiratory syncytial virus, influenza A virus, HIV, measle virus, and dengue virus) favor senescence, most oncogenic viruses (EBV, KSHV, HBV, HCV, and MCPyV) and HCMV have been reported to trigger senescence but also to generate PGCCs [26,143]. Interestingly, HCMV is at the cross-road of senescence and oncogenesis [144], and as for other oncoviruses (e.g., HPV), not all individuals infected with HCMV will develop cancer and non-viral factors (lifestyle and personal immune system) could be also at play. Similar to HPV disease pathophysiology [145], although even healthy individuals might be at risk of HCMV-induced oncogenesis, immunocompromised individuals could develop persistent, treatment-refractory, and progressive HCMV-linked cancers. Following the acute infection of mammary epithelial cells, ovarian epithelial cells, and prostate epithelial cells with high-risk HCMV strains (DB and BL), our team has shown that transformed cells appear in culture including PGCCs [64,68,69] and are tumorigenic in xenografted NSG mice [60], indicating the direct involvement of HCMV in oncogenesis. As we already suggested previously [10], PGCCs should be included in the recently added hallmark of cancer, senescence [8]. 

All in all, HCMV fulfils the recently described four hallmarks of cancer in transformed cells [8], in addition to the previously reported ten hallmarks of cancer [106] (Figure 1).

Since HCMV fulfills all criteria of oncoviruses, this will ultimately pave the way to new therapeutic approaches including antiviral treatments targeting HCMV oncoprotein(s), immunotherapy, and prophylactic vaccination. To assess these new therapeutic strategies, innovative experimental animal models have to be developed such as the generation of glioblastoma in mice engrafted with HCMV-infected astrocytes [94] and the development of triple-negative breast tumors in NOD/SCID Gamma (NSG) mice engrafted with HCMV-infected mammary epithelial cells [60].

## 5. Conclusions

Although HCMV was considered as a herpesvirus with no transforming capacity, twenty years ago, the oncomodulation paradigm emerged that could explain the accelerated progression of cancers fueled by HCMV-infected tumor cells. Recently, a direct transforming role of high-risk oncogenic HCMV strains has been observed which could lead to the appearance of aggressive cancers, with poor prognosis. Based on similar oncogenic features compared to the already recognized seven human oncoviruses and the fulfillment of all the hallmarks of cancer, even the most recent ones described in 2022, the definitive classification of HCMV as the eighth human oncovirus has to be taken into account and will lead to new therapeutic approaches that are actively needed to curtail cancers, especially adenocarcinoma and glioblastoma of poor prognosis.

## Figures and Tables

**Table 1:** Transforming potential of human oncoviruses and HCMV. * HCV core, NS3, NS5A, and NS5B proteins potentiate oncogenic transformation; ** potential transforming HCMV proteins, still to be confirmed.

Viral Agent	Oncoproteins	Cancer Cell Lines/Tumor Types	Associated/Described Outcomes	References
HPV	E6, E7, E5	HPV-positive cervical cancer cells Ca Ski, SiHa HPV16 E6 and E7 expressing esophageal squamous cell carcinoma	Continued proliferation to confluency Lack of apoptosis Reduction in G0/G1 cell cycle arrest PGCCs Establishment of a cancer stem-like phenotype Enhancement of migration, invasion, and spherogenesis Elevated levels of proteins involved in EMT	[27,28,29,30,31]
HBV	HBx, LHBs	HepG2 cells pX-transfected hepatocellular carcinoma cells	Increased tumorigenicity, self-renewal, stemness PGCCs	[32,33,34]
HCV	None (HCV core, NS3, NS5A, NS5B *)		PGCCs	[35,36,37]
HTLV-1	Tax	MT2 and MT4 cells	Increased anti-apoptotic proteins Rb depletion PGCCs	[38,39,40,41,42]
MCPyV	LT-Ag		PGCCs	[43,44,45,46]
EBV	LMP1, EBNA2, EBNA3C, BNRF1	Burkitt’s lymphoma-derived cells LMP1-expressing nasopharyngeal carcinoma cells BHRF1-expressing nasopharyngeal carcinoma cells	Increased cell proliferation Apoptosis suppression Invasion Colony formation ability PGCCs Tumor formation in nude mice	[47,48,49,50,51,52,53]
KSHV	LANA Cyclin K	LANA-expressing human breast cancer cell line MCF7	Inhibition of G2 arrest PGCCs	[54,55,56,57,58,59]
HCMV	IE1, IE2, US28 **	CMV-transformed human mammary epithelial cells (CTH cells), ovarian epithelial cells (CTO cells), prostate epithelial cells (CTP cells), and astrocytes (CEGBCs or CMV-elicited glioblastoma Cells)	Sustained cell proliferation Increased telomerase activity Colony formation ability Stemness EMT Tumor formation in immunodeficient mice	[60,61,62,63,64,65,66,67,68,69]

**Table 2:** Direct oncogenic effect in epithelial cells and astrocytes infected with high-risk HCMV strains results in the generation of transformed CTH, CTO, CTP, and CEGBC cells.

HCMV-Transformed Primary Human Cell	Oncogenic High-Risk HCMV Strains	Phenotypic Features of HCMV-Transformed Cells	Molecular Characteristics of HCMV-Transformed Cells	References
CTH cell (CMV-transformed human mammary epithelial cells)	DB, BL HCMV strains isolated from TNBC tumors	Cellular proliferation of heterogeneous cells PGCCs, giant cell cycling Colony formation in soft agar Dedifferentiation of mature cells during the transformation process Stemness EMT, pEMT Tumor formation in NSG mice	Inactivation of pRb and p53 Activation of telomerase activity Activation of c-Myc, Akt, STAT3 Enhancement of EZH2 Detection of HCMV genes and proteins in transformed cells Reaction of latent virus from transformed cells	[60,61,62,63,64,65,68]
CTO cells (CMV-transformed ovarian epithelial cells)	DB, BL HCMV strains isolated from HGSOC tumors	Cellular proliferation of heterogeneous cells PGCCs, giant cell cycling Colony formation in soft agar Dedifferentiation of mature cells during the transformation process Stemness EMT and pEMT	Inactivation of pRb and p53 Decreased telomerase activity Activation of c-Myc and Akt Enhancement of EZH2 Detection of HCMV genes and proteins in transformed cells	[67]
CTP cell (CMV-transformed prostate epithelial cells)	DB, BL	Cellular proliferation of heterogeneous cells PGCCs, giant cell cycling Colony formation in soft agar Dedifferentiation of mature cells during the transformation process Stemness EMT	Inactivation of pRb and p53 Increased telomerase activity Activation of c-Myc Enhancement of EZH2 Detection of HCMV genes and proteins in transformed cells	[69]
CEGBC (CMV-elicited glioblastoma Cells)	DB, BL HCMV strains isolated from glioblastoma tumors	Cellular proliferation of heterogeneous cells PGCCs Colony formation in soft agar Dedifferentiation of mature cells during the transformation process Stemness EMT Spheroid formation Invasiveness in vitro	Inactivation of pRb and p53 Increased telomerase activity Activation of c-Myc and Akt Enhancement of EZH2 Detection of HCMV genes and proteins in transformed cells	[66]

## Data Availability

Data are contained within the article.
